# Coupling coordination analysis of urbanization and the ecological environment based on urban functional zones

**DOI:** 10.3389/fpubh.2023.1111044

**Published:** 2023-02-03

**Authors:** Xue Luo, Weixin Luan, Yue Li, Tao Xiong

**Affiliations:** ^1^School of Maritime Economics and Management, Dalian Maritime University, Dalian, China; ^2^Faculty of Geosciences and Environmental Engineering, Southwest Jiaotong University, Chengdu, China

**Keywords:** urbanization, sustainable development, coupling coordination degree, RSEI, central Shanghai

## Abstract

Urbanization is an inevitable process in human social progress; additionally, the ecological environment is the carrier and foundation of human social development. Considering central Shanghai, China, as an example, this study quantitatively analyzed the coupling coordination relationship between urbanization and the ecological environment based on urban functional zones; remote sensing images, Open Street Map roads, and point of interest data were analyzed for the urban functional zones *via* the remote sensing-based ecological index (RSEI), comprehensive nighttime light index (CNLI), and coupling coordination degree (D). The results revealed that urban functional zones in central Shanghai were mainly mixed functional zones and comprehensive functional zones, which formed a spatial structure that gradually radiated outward from the urban core. Additionally, CNLI values were high; the proportion of CNLI between 0.6 and 1 was 94.37%. Moreover, the RSEI showed spatial differentiation; it was low in the center and gradually increased outward. Additionally, D was at the primary coordination level. The coupling coordination type in the core area corresponded to an ecological environment lag, which gradually transitioned to a state of systematic balanced development from the core area outward, but showed sluggish urbanization in some areas. This quantitative analysis of the coupling coordination between urbanization and the ecological environment based on urban functional zones provides effective scientific references for optimization of spatial planning.

## 1. Introduction

China has been open to the world since reforms more than 40 years ago. During this period, the urbanization rate has increased from 17.92% in 1978 to 63.89% in 2020 (1). Urbanization is the driving force of socio-economic development and promotes population transformation, industrial restructuring, industrial development, scientific and technological progress, cultural exchange, and more. However, with the advancement of urbanization, highly concentrated populations and the rapid expansion of construction land have led to changes in species richness, climate, vegetation phenology, air quality, water quality, human settlements, and the marine environment (2–15). Ecological and environmental problems have gradually come to the fore and seriously affect the sustainable development of the region. Nevertheless, these issues have gradually become the basic conditions that restrict urban sustainable development. Therefore, analyzing the coupling coordination between urbanization and the ecological environment has become a critical issue in the field of sustainable urban development.

In 2015, the United Nations adopted the 2030 Global Sustainable Development Goals (SDGs), which aim to make cities and human settlements inclusive, safe, resilient, and sustainable (16). In 2021, China's 14th Five-Year Plan explicitly called for a new, improved urbanization strategy that enhances the quality of urbanization development. This new urbanization strategy includes accelerating the citizenship of transferred agricultural populations, improving the spatial layout of urbanization, and comprehensively upgrading the quality of cities (17, 18). Sustainable cities and human settlements focus on sustainable development, and new urbanization prioritizes ecological livability and harmonious development. Therefore, their goals are highly compatible. The relationship between urbanization and the ecological environment is an important element of both sustainable cities and human settlements and new urbanization.

Research on the coupling coordination between urbanization and the ecological environment has mostly focused on geography, urban planning, ecology, environmental science, and economics. Related theories include the environmental Kuznets curve, planetary boundaries theory, tele-coupling theory, sustainable livelihood framework, STIRPAT model, meta-coupling framework, and coupling coordination degree model (19–24). Studies have examined the national, regional, urban agglomeration, provincial, and city scales (19, 25–28). Research methods have included system analyses, mathematical models, and GIS spatial analyses. These studies have mainly focused on the coupling coordination relationships between urbanization and ecological environmental quality, ecological risk, ecological security, ecosystem service value, ecosystem health, geo-ecological environment, and energy efficiency (29–40). Exploring the coupling and coordination relationships between urbanization and the ecological environment can aid in understanding the spatial differentiation and development status of these two factors, which is important for the effective use of geographical location advantages, reasonable urban development planning, and sustainable regional development.

Although previous studies have analyzed the coupling coordination relationship between urbanization and the ecological environment at various levels and scales, certain limitations remain. Most previous studies constructed indicator systems based on socio-economic statistical data, which results in tedious and time-consuming evaluation processes (19). Moreover, the caliber of socio-economic statistics leads to poor spatial resolution of the research results; thus, they cannot reflect coupling coordination relationships at the pixel level (41, 42). Additionally, previous studies have mainly focused on national, regional, urban agglomeration, provincial, and city scales but have failed to express spatial locations and interrelationships of the various functional elements within cities in detail, so that suggestions for optimizing the urban spatial pattern could not be provided (25–27, 41, 42). Furthermore, urbanization in previous studies mainly refers to the urbanization of geographic space, whereas, other studies show that population urbanization provides the largest contribution to urbanization (43). Moreover, few studies have explored the impacts of population urbanization on the ecological environment.

Owing to recent developments, satellite remote sensing has become an effective assessment method for regional urbanization and the ecological environment by remedying defects, such as poor timeliness and low spatial resolution, in socio-economic statistics. Moreover, each city is a complex whole composed of various types of urban functional zones, and the proportion and spatial differentiation characteristic of each functional zone notably affect their operation efficiency, which in turn affects the socio-economic development of the city (44). As the core city of the Yangtze River Delta urban agglomeration, Shanghai has been the most affected by urban diseases and ecological and environmental problems due to its high population density, rapid expansion, severe pollution, and high-risk resource and environmental security threats (45). Therefore, considering central Shanghai as the study area, this study combined population urbanization and geographic space urbanization and quantitatively analyzed the coupling coordination relationship between urbanization and the ecological environment at the pixel level based on urban functional zones.

Overall, this study aimed to accurately identify urban functional zones based on Open Street Map (OSM) road data, point of interest (POI) data, and kernel density estimation methods. Additionally, we examined the spatial patterns of various parameters. The comprehensive nighttime light index (CNLI) was calculated based on nighttime lighting data to reflect the urbanization level throughout geographic space. The remote sensing-based ecological index (RSEI) was calculated based on the normalized difference vegetation index (NDVI), land surface temperature (LST), wetness (WET), and normalized difference bare soil index (NDBSI) to reflect the quality of the regional ecological environment. Furthermore, we aimed to analyze the coupling coordination relationship between urbanization and the ecological environment based on urban functional zones *via* the coupling coordination degree model.

## 2. Materials and methods

### 2.1. Study area

**Figure 1:**
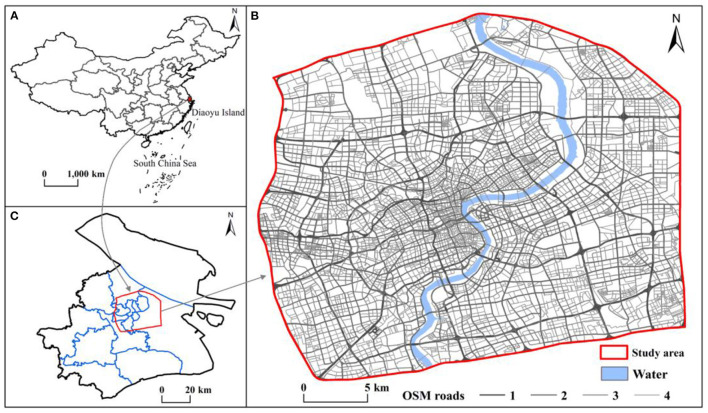
Location of the study area **(A)** China, **(B)** Shanghai, and **(C)** central Shanghai.

The municipality of Shanghai is the international economic, financial, and trade center of China (Figure 1). Shanghai is located on the west coast of the Pacific Ocean, between 105°17′E-110°11′E and 28°10′N-32°13′N. Central Shanghai includes the Huangpu, Xuhui, Changning, Jing'an, Putuo, Hongkou, and Yangpu Districts and the Pudong New Area (within the outer ring only), which is a highly urbanized area with a compact urban spatial form, high population density, economic vitality, and convenient transportation. Therefore, central Shanghai is a typical and representative area for studying the coupling coordination relationship between urbanization and the ecological environment based on urban functional zones.

### 2.2. Data processing

Considering the research goals and data accessibility, the data utilized in this study included OSM road data, POI data, remote sensing image data (Landsat 8 OLI TIRS, NPP-VIIRS, and Worldpop), and administrative division data. The details are shown in Table 1.

**Table 1:** Data sources and descriptions.

**Data types**	**Data descriptions**	**Time**	**Data sources**
OSM	Roads	2020	OpenStreetMap
POI	Point	2020	AMAP Data Open Platform
Landsat 8 OLI TIRS	30 m	2020.07-2020.08	United States Geological Survey
Population	100 m	2020	https://www.worldpop.org
NPP-VIIRS	500 m	2020	https://www.ngdc.noaa.gov
Administrative division	Boundaries	2022	https://www.ngdc.noaa.gov

### 2.3. Methods

#### 2.3.1. Identification of urban functional zones

##### 2.3.1.1. Dividing research units based on OSM road data

OSM data were used to divide the city into different research units. The specific process was as follows: First, based on OSM road classification data, motorway, trunk, primary, secondary, tertiary, residential, and unclassified roads were selected and classified as OSM roads I, II, III, and IV. Road network data was obtained *via* processing the extendline, trimline, and topological check. Subsequently, 40-, 20-, 10-, and 5-m buffers were generated according to the respective road classes, and the buffers were removed from the study area (46, 47). Finally, research units with small areas were discarded to generate the independent research units.

##### 2.3.1.2. Kernel density estimation of POI data

The POI data obtained by AMAP Map has issues such as cross duplication; therefore, it is necessary to eliminate duplicate data based on their attributes. Using the Urban Land Classification and Planning and Construction Land Standard (GB_50137-2011) as the standard, and considering the actual situation in central Shanghai, the POI data were divided into six major categories, namely residential, industrial, commercial, public, science and education, and green square. The division criteria are detailed in Table 2 (48).

**Table 2:** POI data classification.

**Primary classification**	**Secondary classification**	**Quantity**	**Proportion (%)**
Commercial	Shopping services, catering services, accommodation services, leisure, entertainment, and finance	94857	28.78
Green Square	Tourist attractions and park squares	2021	0.61
Industrial	Corporations, industrial and mining plant, and industrial park	94146	25.56
Public	Government agencies, healthcare services, and public facilities	91809	27.86
Residential	Residential area and commercial residential area	21600	6.55
Science and education	Institutions of higher learning, vocational colleges, middle schools, primary schools, scientific, and educational places	25162	7.63

Kernel density estimation (KDE) is an empirical probability density function used to estimate smoothing; its development is based on the first law of geography, which reflects the regularity of spatial location information and distance decay. KDE is widely used in urban public service evaluation, traffic section risk assessment, economic clustering, and other related studies (49–51). It is calculated as follows:


(1) $$\begin{array}{l} \text{f}\left(\text{s}\right)=\overset{n}{\underset{i=1}{\sum }}\frac{1}{h^{2}}\varphi \left(\frac{s-c_{i}}{h}\right) \end{array}$$

where *f*(*s*) is the KDE function located at position *s*, *h* is the attenuation value (bandwidth), *c*_*i*_ is the position of the *i*^*th*^ POI, *n* is the number of POI locations whose path distance from position *s* is not higher than *h*, and φ is the predetermined kernel function. The kernel function has minimal effect on the KDE results, whereas the bandwidth has a notable effect. The larger the bandwidth, the smoother the kernel density surface, masking hot spots in the study area and obscuring their features. When the bandwidth is too small, the kernel density surface becomes uneven, which can reveal fine local features but cannot ensure continuity and correlation in large-scale data, leading to fragmented result patches (52).

##### 2.3.1.3. Identification of urban functional zones

Based on the KDE of the POI data and research units divided by the OSM road data, the frequency density (*F*_*i*_) of each type of POI within each research unit was calculated. Among them, the weights of residential, industrial, commercial, public, science and education, and green square were 30, 40, 15, 50, 60, and 90, respectively (53). The formula for calculating *F*_*i*_ is as follows:


(2) $$\begin{array}{l} F_{i}=\left(W_{i}\;*\;d_{i}\right)/\overset{s}{\underset{j=1}{\sum }}\left(W_{i}\;*\;d_{i}\right)\;*\;100\% \end{array}$$

where *F*_*i*_, *W*_*i*_, and *d*_*i*_ are the frequency density, weight, and kernel density of the *i*^*th*^ type of POI within the research unit, respectively. When the *F*_*i*_ of a POI type was ≥ 50%, the unit was considered as a single type of functional zones; when the *F*_*i*_ of two types of POIs in the research unit were both between 20 and 50%, the research unit was considered as a mixed functional zone of two types. Other units were classified as comprehensive functional zones.

#### 2.3.2. Calculation of CNLI

The CNLI can reflect the intensity of human activity and the level of urbanization in a region. In this study, the CNLI was constructed based on the average relative light intensity (I) and the ratio of the light area to the study area (S) to characterize the level of urbanization (54, 55) as follows:


(3) $$\begin{array}{lll} \text{CNLI} & = & \text{I }*\text{ S} \end{array}$$

(4) $$\begin{array}{lll} I & = & \frac{\sum_{i=P}^{DN_{M}}\left(DN_{i}\;*\;n_{i}\right)}{N_{L}\;*\;DN_{M}} \end{array}$$

(5) $$\begin{array}{lll} S & = & \frac{Area_{N}}{Area} \end{array}$$

Where, *DN*_*i*_ indicates the grayscale value of the *i*^*th*^ image element in the study area, *n*_*i*_ is the total number of the grayscale image elements in the region, *P* is the threshold value for error removal, *DN*_*M*_ is the maximum possible grayscale value, *N*_*L*_ and *Area*_*N*_ are the total number and area of image elements in the region that satisfy the condition *P* ≤ *DN* ≤ *DN*_*M*_, and *Area* is the area of the entire region.

#### 2.3.3. Calculation of RSEI

The RSEI is an index for rapid and objective evaluation of the quality of the regional ecological environment using remote sensing data, which is between 0 and 1. The closer the RSEI is to 1, the better the quality of the ecological environment; the closer the RSEI is to 0, the worse the quality of the ecological environment (56–58). The RSEI was determined by first calculating each component index (NDVI, WET, NDBSI, and LST) separately and standardizing them according to Table 3. Subsequently, the first component of principal component analysis (PC1) and related statistical results were obtained by combining the characteristics of the four indexes; these were normalized to obtain the RSEI.

**Table 3:** Calculation of indicators.

**Index**	**Formula**		**Result**
NDVI	$NDVI=(\rho_{Nir}-\rho_{Red})/(\rho_{Nir}+\rho_{Red})$	(6)	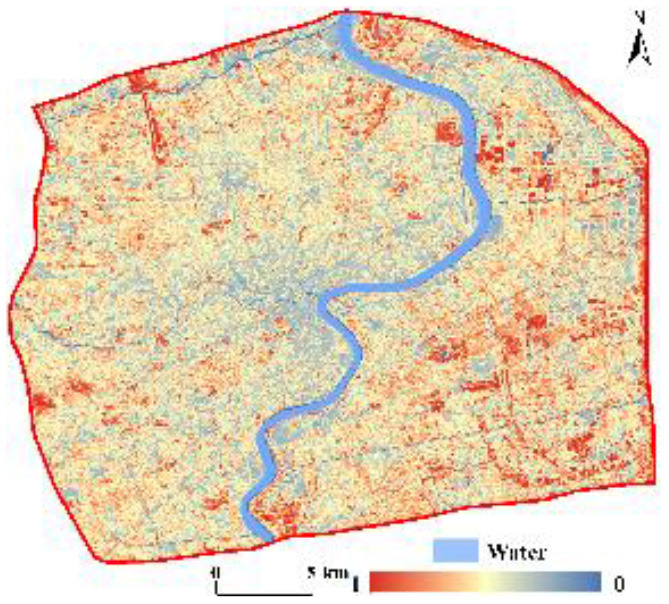
WET	$\begin{array}{l} WET=0.1511\;*\;\rho_{Blue}+0.1973\\ \;*\;\rho_{Green}+0.3283\\ \;*\;\rho_{Red}+0.3407\;*\;\rho_{Nir}\\ \;-0.7117\\ \;*\;\rho_{Swir1}-0.4559\;*\\ \;\rho_{Swir2} \end{array}$	(7)	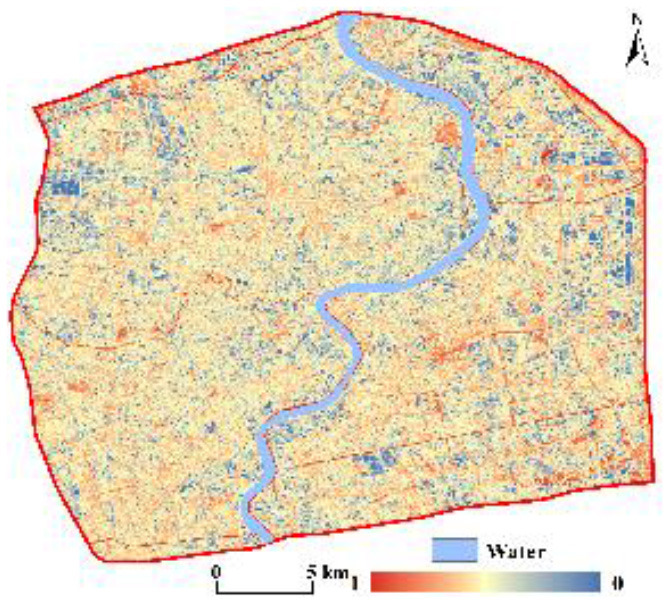
NDBSI	$\begin{array}{l} IBI=\{\frac{2\rho_{Swir1}}{\left(\rho_{Swir1}+\rho_{Nir}\right)}\\ \;-[\frac{\rho_{Nir}}{\left(\rho_{Nir}+\rho_{Red}\right)}\\ \;+\frac{\rho_{Green}}{\left(\rho_{Green}+\rho_{Swir1}\right)})]\}\\ \;/\{\frac{2\rho_{Swir1}}{\left(\rho_{Swir1}+\rho_{Nir}\right)}\\ \;+[\frac{\rho_{Nir}}{\left(\rho_{Nir}+\rho_{Red}\right)}\\ \;+\frac{\rho_{Green}}{\left(\rho_{Green}+\rho_{Swir1}\right)}]\}\;\\ SI=\frac{\left(\rho_{Swir1}+\rho_{Red})-(\rho_{Nir}+\rho_{Blue}\right)}{\left(\rho_{Swir1}+\rho_{Red})+(\rho_{Nir}+\rho_{Blue}\right)}\;\\ \;NDBSI=(IBI+SI)/2\; \end{array}$	(8) (9) (10)	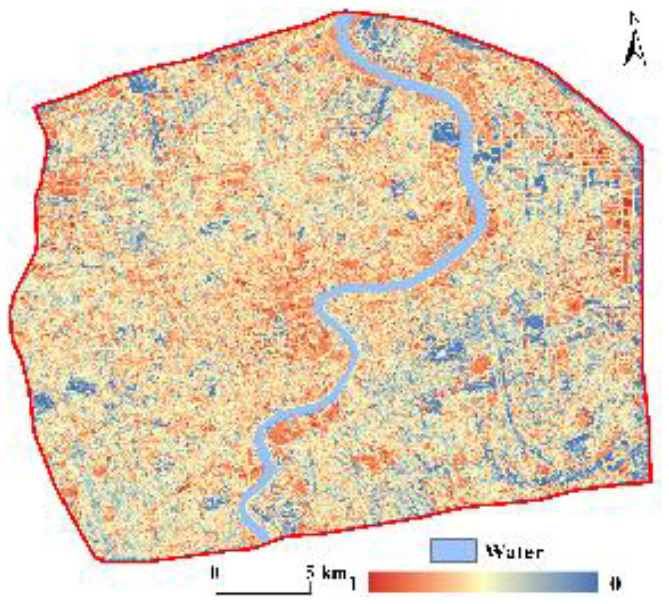
LST	$\begin{array}{l} T_{s}=[a\;*\;\left(1-C-D\right)\\ \;+(b\;*\;(1-C-D)\\ \;+C+D)\;*\;T_{i}-D\;*\\ \;T_{a}]/C\\ \;C=\varepsilon \;*\;\tau \;\\ \;D=\left(1-\tau \right)\;*\;[1+(1-\varepsilon )\;*\;\tau ]\; \end{array}$	(11) (12) (13)	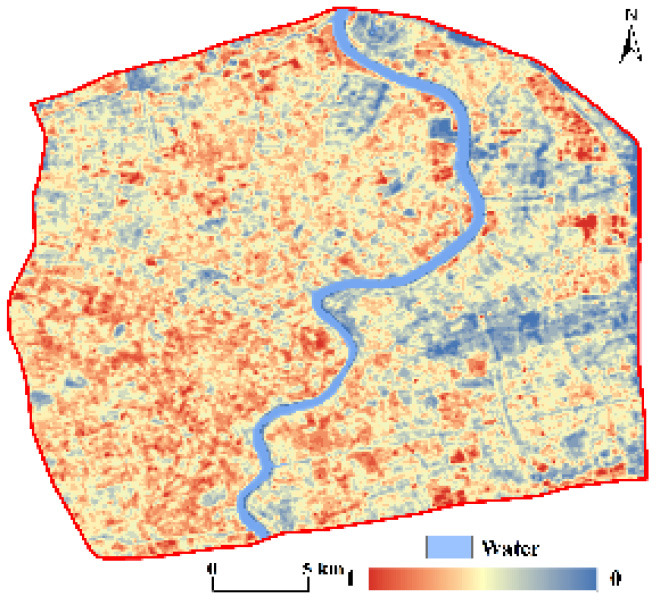

In this table, ρ_*Red*_, ρ_*Green*_, ρ_*Blue*_, ρ_*Nir*_, ρ_*Swir*1_, and ρ_*Swir*2_ correspond to each band of the Landsat 8 OLI TIRS; *T*_*s*_ is the land surface temperature; *T*_*a*_ is the atmospheric temperature, which is generally constant and was taken as 293.16 in this study; *T*_*i*_ is the brightness temperature of thermal infrared band; *a* and *b* are constants and are−67.355 and 0.459, respectively; ε is the surface specific emissivity; and τ is the atmospheric transmittance.

#### 2.3.4. Coupling coordination degree (D)

The D can characterize the interaction of two or more systems and their coordination degree. It is mainly used to analyze coordination levels and has widely been used in the study of the coupling coordination relationships among urbanization, social economy, the ecological environment, and industrial structure (59–65). Urbanization includes both geospatial urbanization and population urbanization. Geospatial urbanization provides support and assurance for urbanization, and population urbanization is the essence of urbanization. In this study, the CNLI and population represented geospatial and population urbanization, respectively. To explore the coupling coordination relationship between urbanization and the ecological environment, we integrated the CNLI and population at equal proportions (i.e., 0.5 each) into the urbanization system and built the urbanization index. The formula for the D is as follows:


(6) $$\begin{array}{lll} C & = & {\left\{\frac{U_{1}*U_{2}}{{\left[\frac{\left(U_{1}+U_{2}\right)}{2}\right]}^{2}}\right\}}^{\frac{1}{2}} \end{array}$$

(7) $$\begin{array}{lll} T & = & \alpha *U_{1}+\beta *U_{2} \end{array}$$

(8) $$\begin{array}{lll} D & = & \sqrt{C^{*}T} \end{array}$$

where *C* is the coupling degree; *U*_1_ and *U*_2_ are the index values of urbanization and the ecological environment, respectively; *T* is their comprehensive evaluation value; and α, β are coefficients. We considered that urbanization and the ecological environment were equally important; therefore, α = β = 0.5. The *D* is the coupling coordination degree; the larger the *D*, the better the coupling coordination degree and the more coordinated the development level between urbanization and the ecological environment.

## 3. Results

### 3.1. Urban functional zones

**Figure 2:**
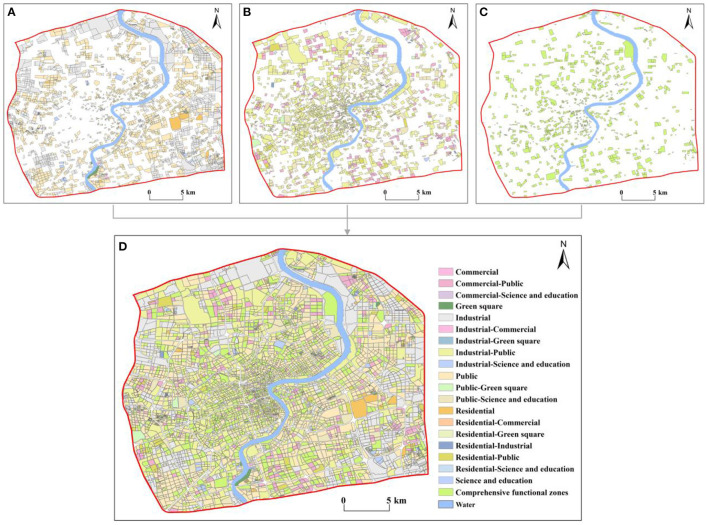
Classification of urban functional zones in central Shanghai **(A)** single-type functional zones, **(B)** two-type mixed functional zones, **(C)** comprehensive functional zones, and **(D)** all functional zones within central Shanghai.

Based on the OSM road network data, central Shanghai was divided into 6,219 research units and 20 types of urban functional zones, including six single-type, 13 two-type mixed, and one comprehensive functional zone types (Figure 2). The spatial distribution of the single-type functional zones is shown in Figure 2A. Public functional zones were the most numerous among these, at 1,666 units, which accounted for 26.79% of the total units. Figure 2B shows the two-type mixed functional zones, the most numerous of which were industrial-public functional zones, with 1,546 instances, which comprised 24.86%. The comprehensive functional zones totaled 944, accounting for 15.18% (Figure 2C). Overall (Figure 2D), mixed functional zones and comprehensive functional zones were predominant in central Shanghai, accounting for 54.91% of the total units. These zones gradually decreased from the center of the city to the perimeter, forming a spatial structure that gradually radiates outward from the core of the city; this indicates that the distribution of urban functional zones was reasonable, which helps improve the efficiency of urban operation and enhance urban vitality. The single-type functional zones were scattered throughout the core of the city but gradually clustered outward; this was especially true of the industrial functional zones, which were mainly concentrated in the Gaoqiao and Shanghai New Cao Yang industrial parks.

### 3.2. Spatial patterns of the parameters

#### 3.2.1. Comprehensive nighttime light index

**Figure 3:**
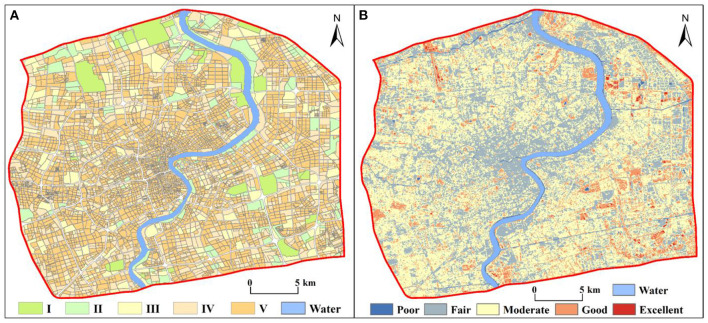
Spatial patterns of the CNLI **(A)** and RSEI **(B)** in central Shanghai.

The CNLI can reflect the urbanization level of a region, which we utilized to reveal the urbanization levels in central Shanghai from the perspective of urban functional zones. Utilizing ArcGIS, the CNLI was divided into five classes: I (0–0.6), II (0.6–0.7), III (0.7–0.8), IV (0.8–0.9), V (0.9–1.0). The results are shown Figure 3A. The CNLI was high in the core of the city (with an average value of 0.92) and gradually decreased toward the periphery; simultaneously, it had good continuity, which indirectly reflects the close connection between social and economic activities and population flow within these areas. The percentages of the II, III, IV, and V classifications were 7.51, 13.42, 26.20, and 47.24%, respectively.

#### 3.2.2. Sensing-based ecological index

The RSEI ranged from 0 to 1 and, overall, was low in the center of the city and gradually increased toward the periphery. The RSEI was also divided into five classes using the equal interval method in ArcGIS as follows: poor (0–0.2), fair (0.2–0.4), moderate (0.4–0.6), good (0.6–0.8), and excellent (0.8–1). Figure 3B shows that the RSEI in central Shanghai was primarily between 0.4 and 0.6; thus, the ecological environment was in moderate condition. The RSEI in the core of the city was mostly between 0.2 and 0.4; therefore, the ecological environment was fair in the city core. The ecological environment quality in the periphery of the central city was notably better than that in the core area, especially in some areas in southeastern central Shanghai, where the ecological environment was in excellent condition. Additionally, the area and proportion of the five classes were separately calculated. The area in the moderate class was 225.05 km^2^, comprising 51.39% of the area; the percentages of the areas of the remaining classes in descending order were as follows: fair (33.86%) > good (11.15%) > poor (3.16%) > excellent (0.44%).

### 3.3. Spatial pattern of coupling coordination degree (D)

**Figure 4:**
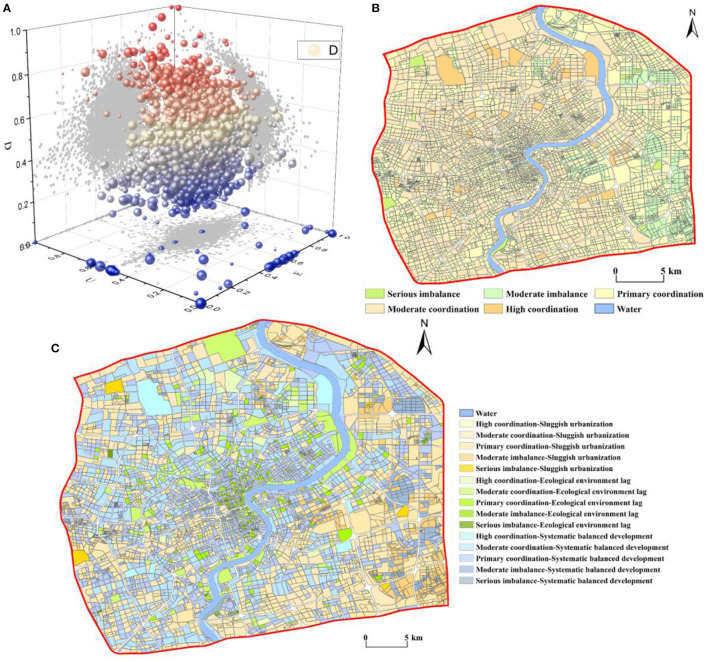
Spatial pattern of the D in central Shanghai **(A)** 3D scatter plot of D, **(B)** classes of D, and **(C)** coupling coordination classifications.

The D was between 0 and 1 (Figure 4A) and was classified into five classes using the equal interval method of ArcGIS (Figure 4B) as follows: serious imbalance (0–0.2), moderate imbalance (0.2–0.4), primary coordination (0.4–0.6), moderate coordination (0.6–0.8), and high coordination (0.8–1), which accounted for 2.46, 23.32, 54.73, 18.69, and 0.80% of research units, respectively. The mean D value in central Shanghai was 0.48, which indicates a primary coordination level; this is closely related to the high urbanization level and high population density in the region. In terms of the spatial heterogeneity of the D, the core of Shanghai was generally in a state of primary coordination. The D gradually increased from the core area outward and gradually transitioned to the moderate coordination level. In the eastern areas of central Shanghai, moderate imbalance and primary coordination were predominant, indicating that the D between urbanization and the ecological environment was relatively low in this region. Nevertheless, some areas showed a high degree of coordination, such as Gongqing National Forest Park and Tongji University, which are mostly science and education, green square, or public functional zones. Although the five types of the D showed dispersed spatial differentiation, there were also clustering phenomena in some areas, such as in a newly developed industrial park, which was within the moderate imbalance level.

We integrated the CNLI and population with 0.5 to create a unified urbanization index (U). As shown in Table 4, these values were combined with the RSEI (E) to classify the specific types of coupling coordination into three major categories: ecological environment lag, sluggish urbanization, and systematic balanced development. The results show that within central Shanghai, the main category was systematic balanced development; the percentages of areas with ecological environment lag, sluggish urbanization, and systematic balanced development were 10.53, 37.28, and 52.20%, respectively.

**Table 4:** Classification categories for the coupling coordination of urbanization and the ecological environment.

**Composite category**	**Coordination level**	**Subcategory**	**Systematic exponential comparison**	**Subcategory**	**Percent (%)**
Coordinated development	0.8 < D ≤ 1	High coordination	E-U > 0.1	Sluggish urbanization	0.10
			|E-U| ≤ 0.1	Systematic balanced development	0.21
			E-U < −0.1	Ecological environment lag	0.50
Transformation development	0.6 < D ≤ 0.8	Moderate coordination	E-U > 0.1	Sluggish urbanization	6.31
			|E-U| ≤ 0.1	Systematic balanced development	10.32
			E-U < −0.1	Ecological environment lag	2.07
	0.4 < D ≤ 0.6	Primary coordination	E-U > 0.1	Sluggish urbanization	20.67
			|E-U| ≤ 0.1	Systematic balanced development	29.32
			E-U < −0.1	Ecological environment lag	4.75
Uncoordinated development	0.2 < D ≤ 0.4	Moderate imbalance	E-U > 0.1	Sluggish urbanization	9.03
			|E-U| ≤ 0.1	Systematic balanced development	11.60
			E-U < −0.1	Ecological environment lag	2.68
	0 < D ≤ 0.2	Serious imbalance	E-U > 0.1	Sluggish urbanization	1.17
			|E-U| ≤ 0.1	Systematic balanced development	0.75
			E-U < −0.1	Ecological environment lag	0.53

Combined with the classification of coupling coordination shown previously, the specific types of coupling coordination were further classified into 15 categories. As shown in Table 4, the proportion of areas with primary coordination-systematic balanced development was the highest, reaching 29.32%; this was followed by primary coordination-sluggish urbanization at 20.67%. Only two types had a percentage between 10 and 20%, namely moderate imbalance-systematic balanced development and moderate coordination-systematic balanced development; all other specific types of coupling coordination were below 10%. Figure 4C shows the spatial characteristics of the specific types of coupling coordination; the predominant type of coupling coordination in the core of central Shanghai was ecological environment lag. However, this gradually transitioned to a state of systematic balanced development from the core area outward but showed sluggish urbanization in some areas of Pudong New Area, Baoshan District, and Changning District. In general, this was consistent with the actual urbanization and ecological situation of central Shanghai.

## 4. Discussion

### 4.1. Why utilize the perspective of urban functional zones?

Accurate identification of urban functional zones plays a significant role in spatial planning and sustainable development decisions at various scales (66). The coupling coordination between urbanization and the ecological environment is an important element of urban sustainable development (67). This study examined this relationship through the perspective of urban functional zones because existing studies have mostly constructed indicator systems based on socio-economic statistics, which have poor precision. Moreover, the research scales of previous studies have mostly been based on countries, regions, urban agglomerations, provinces, and cities; thus, they cannot directly reflect spatial differences in the coupling coordination relationship between urbanization and the ecological environment within cities (19, 25–27). Additionally, urban roads are the basis for urban development and lead to further development; moreover, they have far-reaching influences on socio-economic development. Hence, the parcels formed by urban road connections are the basic units of urban planning and give rise to urban socio-economic functions. The OSM road data have high positioning accuracy and good topological relationships and are widely used in network and service-zone analysis (68), thereby enabling accurate identification of urban functional zones. Overall, multi-source data are more reasonable for quantitatively exploring the coupling coordination relationship between urbanization and the ecological environment within cities based on urban functional zones.

In summary, we divided the study area into research units based on OSM road data and identified urban functional zones based on POI data. Subsequently, the CNLI and RSEI were calculated based on remote sensing image data to quantitatively analyze the coupling coordination relationship between urbanization and the ecological environment within each urban functional zone. This method overcomes the limitations of previous studies, considers the urban spatial structure composed of urban roads, and expresses the coupling coordination at a fine scale. Moreover, exploring the coupling coordination relationship between urbanization and the ecological environment within a city provides effective scientific references for formulating urban development strategies and optimizing spatial planning; it can also effectively promote socio-economic development and construction of an ecological civilization.

### 4.2. Differences among various types of urban functional zones

**Figure 5:**
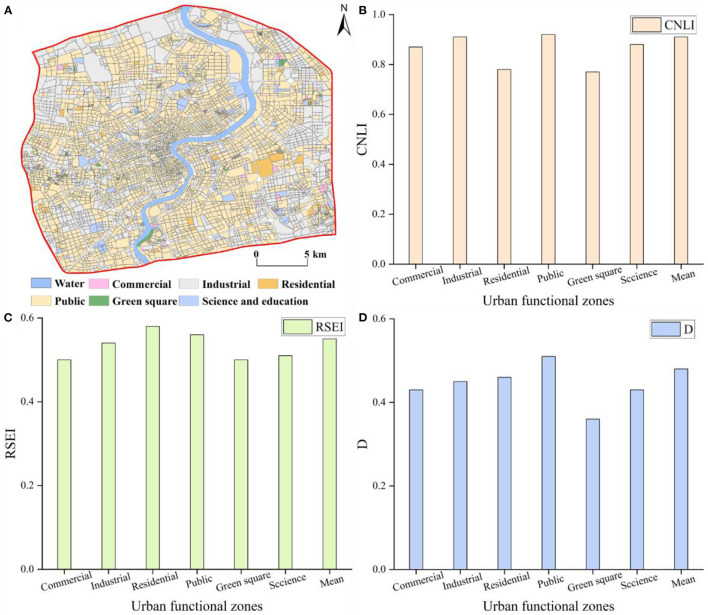
Differences in indices in various urban functional zones **(A)** functional zones by dominant type, **(B)** CNLI, **(C)** RSEI, and **(D)** D.

The refined urban functional zones (Figure 2) were categorized into commercial, industrial, residential, public, green square, and science and education functional zone types. As shown in Figure 5A, public-dominated functional zones were predominant in central Shanghai, accounting for 64.91% of the total units, followed by industrial-dominated functional zones at 29.18% of the total. Figure 5B shows differences in the CNLI among functional zones. The CNLI was high throughout; notably, the average CNLI value in public and industrial-dominated functional zones was above 0.90. The mean RSEI value was ~0.50; the RSEI was higher in residential- and public-dominated functional zones (Figure 5C). Figure 5D shows differences in the D among various types of functional zones. The D of green square-dominated functional zones was the lowest, whereas the D was the highest in public-dominated functional zones. The D in the remainder of functional zones decreased from residential- to industrial-, science and education-, and commercial-dominated functional zones.

**Figure 6:**
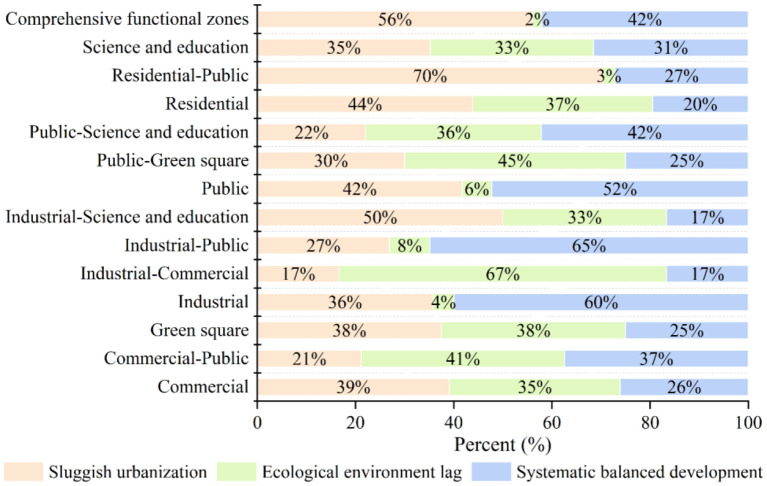
Percentage of coupling coordination classifications in each urban functional zone.

The percentages of ecological environment lag, sluggish urbanization, and systematic balanced development in the different types of urban functional zones were calculated to reveal spatial patterns in the D. The results are shown in Figure 6; note that functional zone types with low numbers were removed from the calculation. The commercial, green square, industrial-science and education, residential, residential-public, science and education, and comprehensive functional zones primarily showed sluggish urbanization. In contrast, ecological environment lag was predominant in commercial-public, industrial-commercial, and public-green square zones. Additionally, industrial, industrial-public, public, and public-science and education zones mainly showed systematic balanced development. Although comprehensive functional zones predominantly showed sluggish urbanization, 42.06% of these zones showed systematic balanced development, indicating that their urbanization was nevertheless coordinated with the development of the ecological environment.

### 4.3. Recommendations for urban planning

The United Nations Sustainable Development Goals (SDGs) propose that cities and human settlements should be inclusive, safe, resilient, and sustainable. Similarly, new urbanization is an urbanization process that harmonizes the population, economy, resources, and environment and emphasizes improving the capacity for sustainable urban development (69). The coupling coordination between the development of urbanization and the ecological environment is the core issue for both SDGs and new urbanization. Central Shanghai is the political and economic center of the city and has a high level of urbanization and a highly aggregated population. As urbanization continues to advance in Shanghai, the pressure on the ecological environment will continuously increase. Therefore, promoting high-quality development *via* new urbanization and construction of an ecological civilization is a long-term test for central Shanghai.

Research reveals that the impact of urbanization on coupling coordination is 7.2 times higher than that of the ecological environment; therefore, to improve the coupling coordination relationship between urbanization and the ecological environment, increasing the quality of urbanization is important (42). Territorial spatial planning provides a spatial blueprint for urban sustainable development and integrates the functions and characteristics of spatial planning; thus, it is an important tool to improve the quality of urbanization (70). In the context of the COVID-19 epidemic, new urbanization and the construction of sustainable cities and human settlements should be set as the goal; territorial spatial planning considered as the method; urban functional zones used as the scale; the rational allocation and renewal of the urban infrastructure and public service facilities optimized; the coupling coordination relationship between urbanization, population, and the ecological environment improved; urban ecological resilience enhanced; and sustainable development promoted.

In this study, the coupling coordination relationship in the core area of central Shanghai primarily manifested as ecological environment lag, indicating that its ecological spatial pattern should be further optimized; simultaneously, public awareness of ecological environmental protection should be further strengthened to enhance the environmental carrying capacity and improve regional ecological and environmental benefits. The coupling coordination relationship between urbanization and the ecological environment in some areas at the periphery of central Shanghai showed sluggish urbanization. The urbanization process in these areas should be appropriately strengthened and its quality actively improved. Additionally, the population flow should be reasonably guided, such that the population level can be gradually raised to alleviate the pressure on the ecological environment caused by high population density and traffic congestion in the core urban area. While accelerating urbanization, we should also consider protecting the ecological environment by implementing precise monitoring and management policies and maintaining the steady progress of ecological civilization construction. Moreover, enterprises should increase their investment in green development, thereby improving the overall environmental quality of the city and realizing sustainable development in the region.

### 4.4. Limitations

The exploration of the coupling coordination relationship between urbanization and the ecological environment based on urban functional zones has certain limitations. OSM road data have high positioning accuracy and good topological relationships; however, in their application to urban functional zone delineation, the grades used have an influence on the final delineation results. In future study, we will combine OSM data with high-resolution remote sensing image data to improve the precision of the delineation, as well as its consistency with the spatial structure and development pattern of the city (71). Moreover, we only analyzed the coupling coordination relationship between urbanization and the ecological environment in central Shanghai, which is small in scale. In the future, this relationship can be analyzed for urban agglomerations and other larger areas. The impact of population urbanization on the ecological environment can also be further refined to provide solid theoretical support for the implementation of national strategies, such as ecological environmental protection and high-quality development (72).

## 5. Conclusions

We examined central Shanghai, China, *via* GIS spatial analysis and KDE using multi-source data, such as remote sensing images, OSM roads, and POIs, and analyzed the spatial differentiation of the coupling coordination relationship between urbanization and the ecological environment based on urban functional zones. The main conclusions of this study are as follows: Central Shanghai had a high urbanization level and a moderate ecological environment quality. Additionally, the D mainly indicated a primary coordination level, showing a gradual increase to a moderate coordination level from the core area outward. The specific coupling coordination types mainly showed a systematic balanced development; ecological environment lag, sluggish urbanization, and systematic balanced development were found in 10.53, 37.28, and 52.20% of study units, respectively. At the same time, different types of urban functional zones had different coupling coordination relationships. industrial, industrial-public, public, and public-science and education zones mainly showed systematic balanced development.

This study examined the relationship between urbanization and the ecological environment from the perspective of urban functional zones, which is conducive to understanding the spatial differentiation and development status of urbanization and the ecological environment within cities. This perspective not only ensures a complete and accurate urban spatial structure and plot size, but also avoids overly fragmented divisions, providing a new perspective for related research. Moreover, with reference to the limitations of previous research, exploring the coupling coordination relationship between urbanization and the ecological environment based on the perspective of urban functional zones also provides a breakthrough idea for future generations to perform similar research. Additionally, this study emphasizes the importance of population urbanization in the urbanization process, which is important for optimizing the spatial distribution of populations and urban planning. In subsequent research, we will integrate multi-source data, such as high-resolution remote sensing image data, to enhance the recognition accuracy for urban functional zones and compare the coupling coordination relationship between urbanization and the ecological environment at multiple scales to provide theoretical support for hierarchical and classified territorial spatial planning.

## Data availability statement

The original contributions presented in the study are included in the article/supplementary material, further inquiries can be directed to the corresponding author.

## Author contributions

WL: all aspects of this study. XL: investigation, analysis, validation, writing – original draft, and writing – review and editing. YL: writing – review and editing. TX: identification of urban functional zones and writing – review and editing. All authors contributed to the article and approved the submitted version.
